# Application of the Brillouin Optical Scanning System in the Regional Corneal Biomechanical Evaluation of Keratoconus and Its Correlation with Corvis ST Parameters

**DOI:** 10.3390/bioengineering12060634

**Published:** 2025-06-11

**Authors:** Qiuruo Jiang, Yichen Sun, Zhanhao Gu, Lumeng Wang, Yiqiang Wu, Jialu Chen, Zhiyi Chen, Xiaobo Zheng, Shihao Chen

**Affiliations:** 1 National Clinical Research Center for Ocular Diseases, Eye Hospital, Wenzhou Medical University, Wenzhou 325027, China; jiangqr@mail.eye.ac.cn (Q.J.); ycs1229@163.com (Y.S.); gzh3121@163.com (Z.G.); lulukiowww@163.com (L.W.); 18905488406@163.com (Y.W.); cjl13676718612@163.com (J.C.); 2 National Engineering Research Center of Ophthalmology and Optometry, Eye Hospital, Wenzhou Medical University, Wenzhou 325027, China; 3 State Key Laboratory of Ophthalmology, Optometry and Visual Science, Eye Hospital, Wenzhou Medical University, Wenzhou 325027, China; 4 Institute of Education Faculty, Psychology and Human Development Department, University College London, London WC1E 6BT, UK; qtnzzch@ucl.ac.uk

**Keywords:** corneal biomechanical, keratoconus, Corvis ST, Brillouin optical scanning system

## Abstract

(1) Background: The early diagnosis of keratoconus is critical for prognosis. Traditional methods like ORA and Corvis ST measure overall corneal biomechanics but lack regional specificity and are affected by intraocular pressure. In contrast, Brillouin microscopy assesses regional corneal biomechanics without such limitations; (2) Methods: In total, 25 keratoconus patients and 28 healthy controls were included in this study. Corneal biomechanics were measured using the BOSS system (Brillouin Optical Scanning System) in a 10-point mode within an 8 mm diameter, and included the mean, maximum, minimum and standard Brillouin shift. The Corvis ST parameters extracted included the CBI (Corneal Biomechanical Index), CCBI (Corvis Biomechanical Index for Chinese populations), SSI (Stress–Strain Index), DA (Deformation Amplitude), IIR (Inverse Integrated Radius), and SP-A1 (Stiffness Parameter at First Applanation); (3) Results: BOSS showed significant differences in the inferior nasal region (*p* = 0.004) and central region (*p* = 0.029) between groups, but not in peripheral regions (*p* = 0.781). In a comparison of the Brillouin frequency shifts measured between groups, there was no difference in the Mean (*p* = 0.452) and Max (*p* = 0.487), but the Min (*p* = 0.003), Standard (*p* = 0.000), and Max–Min (*p* = 0.006) all showed differences. Corvis ST identified significant differences in six parameters (CBI, CCBI, SSI, DA, IIR, and SP-A1) between groups (*p* < 0.001). Correlations were found between the BOSS and Corvis ST results, with moderate correlations in the inferior nasal region; (4) Conclusions: The BOSS Brillouin microscope can provide an accurate diagnostic evaluation for the corneal biomechanical differences between normal eyes and keratoconus, independent of IOP (Intraocular Pressure) and CCT (Central Corneal Thickness), with a good correlation with Corvis ST, especially in assessing regional biomechanics.

## 1. Introduction

Keratoconus is a non-inflammatory, progressive corneal ectatic disease characterized by weakened local biomechanical properties in the cornea and thinning and protrusion in the central or paracentral area, leading to irregular astigmatism and vision loss. In severe cases, corneal transplantation is required to restore visual function [1,2,3]. The early diagnosis of keratoconus is crucial for the prognosis of patients and is also a challenge in current research and clinical practice [4,5,6]. Studies have shown that the occurrence of keratoconus is mainly due to a decline in the regional biomechanical properties of the cornea rather than the weakening of the overall uniform biomechanical properties [7,8]. Therefore, achieving the in vivo measurement of the regional corneal biomechanical properties of the human eye and obtaining the microscopic and regional biomechanical distribution patterns of the cornea is of great value in improving the diagnosis rate of keratoconus [9,10].

At present, only the ocular response analyzer (ORA; Reichert Ophthalmic Instruments, Depew, NY, USA) and Corvis ST (CST; Oculus Optik-gerate, Inc., Wetzlar, Germany) are available for the in vivo measurement of the biomechanical properties of the human cornea [2,11,12]. Both ORA [13] and CST [14] assess corneal biomechanical properties by using rapid and large air pulses as the excitation force, which cause deformation of the cornea both internally and externally. The deformation of the cornea is recorded by a photoelectric system (ORA) and Scheimpflug imaging system (CST). However, both can only reflect the overall biomechanical properties of the cornea to a certain extent and cannot measure the biomechanical properties of the cornea in different regions. Moreover, their measurement results are all affected to some extent by intraocular pressure, which limits their application in the early diagnosis of keratoconus [14,15,16]. Brillouin microscopy is a high-resolution, non-contact, and non-perturbing all-optical imaging technique. It can directly evaluate the biomechanical properties of the cornea through the relationship between the Brillouin frequency shift and longitudinal elastic modulus [17]. Unlike traditional measurement methods that rely on the macroscopic deformation of the cornea, Brillouin technology can provide local mechanical information about the cornea without interference from intraocular pressure and corneal geometric factors [18,19]. This makes it show unique potential in the diagnosis of keratoconus and the assessment of local pathological changes [8]. However, previous studies have shown that Brillouin microscopy can effectively evaluate keratoconus at different stages [18,20]. At the same time, regional measurements have also been conducted on patients with subclinical keratoconus (SKC) [21], but research on the regional biomechanical distribution of the cornea remains blank.

This study aims to evaluate the regional distribution patterns of the biomechanical properties of normal corneas and keratoconus corneas through a novel Brillouin optical scanning system (BOSS; Intelon Optics, Woburn, MA, USA), and to explore the correlations between these properties and Corvis ST parameters; this is in order to provide crucial evidence for enhancing the diagnostic rate of keratoconus and formulating optimized treatment strategies.

## 2. Materials and Methods

### 2.1. Subjects

This study is a prospective one. A total of 25 patients with clinical keratoconus (KC group) and 28 eyes from 28 normal individuals (healthy control group) who visited the refractive surgery center of Eye Hospital of Wenzhou Medical University from July 2023 to July 2024 were included. In accordance with the principles of the Helsinki Declaration, all patients signed written informed consent forms.

All patients underwent comprehensive ophthalmic examinations, including intraocular pressure measurement, slit lamp examination, subjective refraction, fundus examination, and corneal topography (Pentacam HR; Oculus Optikgerate GmbH, Wetzlar, Germany) examination.

Wearing soft contact lenses had to be discontinued for at least 2 weeks and wearing RGP lenses had to be discontinued for 1 month before the examination. The inclusion criteria were as follows: (1) KC group: Patients diagnosed with keratoconus in both eyes by two senior doctors, with no previous history of any ocular surgery (including corneal cross-linking). (2) Normal group: No abnormalities in both eyes, including on normal slit lamp biomicroscopy examination, a corrected distance visual acuity greater than or equal to 20/20, and no abnormal signs on corneal topography. The exclusion criteria included any previous history of ocular surgery or disease.

### 2.2. Corneal Biomechanical Measurement

#### 2.2.1. BOSS

BOSS is a high-precision optical instrument based on laser technology, consisting of a near-infrared single-wavelength diode laser source and a Brillouin spectrometer (employing a two-stage “virtual imaging phased array” design). Wavelength: 780 ± 1 nm, Measurement Spatial Range: Lateral (corneal measurements): ±5 mm. Measurement Range: Cornea: 2.30–3.08 GPa. According to the IEC 60825-1 Laser Class standard [22], the laser energy is Class 1, and the calculated exposure amount for different eye parts under different stimulation conditions is within the maximum permissible exposure (Maximum Permissible Exposure (MPE)) range. The laser output power is less than 40 mW. The collimated beam in the reference arm is focused into a small sample cell containing water and polystyrene plastic. The light focus is located at the interface between water and plastic, enabling the simultaneous acquisition of the Brillouin signals of both materials. The backscattered light is guided to the spectrometer to precisely calculate the Brillouin frequency shift, and then the elastic modulus of the two materials is derived based on the calibration signal. Before the formal measurement, the instrument accuracy is ensured through calibration; during the measurement process, the system first activates the reference arm to complete the calibration, and then switches to the sample arm to scan the eye area of the subject.

**Figure 1:**
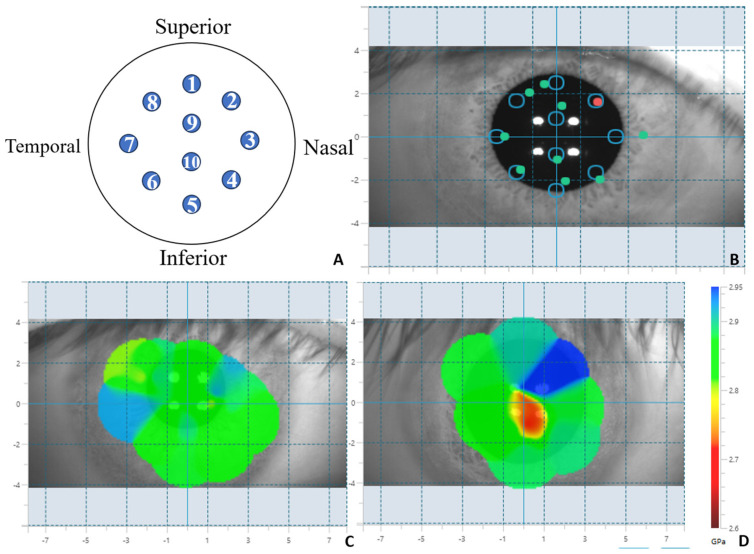
BOSS system measurement mode and distribution map. (**A**): BOSS ten-point measurement mode position diagram of measured points; (**B**): BOSS ten-point mode collection pattern (the circle-marked positions are the target measurement locations, while the green and red dots are the actual measurement positions); (**C**): Distribution map of Brillouin modulus of cornea in normal individuals; (**D**): Distribution map of Brillouin modulus of keratoconus.

During the process of measuring the Brillouin frequency shift modulus, the subjects were seated, with their chin and forehead fixed on the jaw support and forehead support, respectively. The subjects were instructed to fix their gaze on the fixation target. This study adopted a measurement mode with a diameter of 5 mm and 10 points (as shown in Figure 1A,B). The BOSS system has a passive eye movement tracking function. The operator aligns the central crosshair with the center of the pupil. The system automatically calibrates and records the target position and actual scanning position of each measurement point through real-time pupil detection and tracking, and marks them on the output graph (as shown in Figure 1B: the circles mark the target measurement positions, and the green and red dots mark the actual measured positions). The data from the 10-point measurement mode were divided into four directions: nasal up (the average of points 1, 2, and 3), nasal down (the average of points 3, 4, and 5), temporal up (the average of points 1, 7, and 8), and temporal down (the average of points 5, 6, and 7). These data were also divided into two regions: central (the central area value directly given by the system) and peripheral (the average of points 1 to 8).

BOSS generates a two-dimensional Brillouin distribution map based on the measured values (as shown in Figure 1C,D). Based on the two-dimensional distribution map, BOSS extracts the maximum Brillouin frequency shift (Max), the minimum Brillouin frequency shift (Min), the average Brillouin frequency shift in this point (Mean), the standard deviation of spatial distribution (St. Dev), and the Max–Min Brillouin frequency shift value. The Brillouin frequency shift values derived by BOSS are in GPa units, and the conversion formula from GHz is as follows:

(1) $$\mathrm{Brillouin}\;\mathrm{shift}\;(\Omega )=\sqrt{\frac{4M\times 10^{9}}{570\times ({{780\times 10}^{-9})}^{2}}\times 10^{-9}}$$

M represents the frequency of the Brillouin scattering light. We can determine the frequency shift by measuring the frequency of the Brillouin scattering light, and thereby obtain the elastic properties of the material, namely the Brillouin modulus. We adopt the 10-points measurement method and use the system’s own algorithm to obtain the central Brillouin modulus. We manually extract the data of the surrounding 8 points and calculate the average Brillouin modulus of the surrounding area, as well as the Brillouin modulus of each zone in the surrounding area.

#### 2.2.2. Corvis ST

Corvis ST employs an ultra-fast Scheimpflug camera to dynamically observe corneal deformation responses in a 5–6 mm area during a jetting process. Once the measurement is completed, the device provides a set of corneal deformation parameters based on the dynamic examination of corneal responses. This study only included visual corneal biomechanical analyzers with a quality score of “OK” for the visual corneal biomechanical analysis, taking into account the parameters that reflect the biomechanical properties of the cornea for analysis, including CBI (Corneal Biomechanical Index), CCBI (Corvis Biomechanical Index for Chinese populations), SSI (Stress–Strain Index), IIR (Inverse Integrated Radius), DA (Deformation Amplitude), and SP-A1 (Stiffness Parameter at First Applanation).

#### 2.2.3. Statistical Analysis

The data were processed by using SPSS statistical software (version 27). The normality of the data was tested by the Shapiro–Wilk test. The independent sample T test was used for comparisons between the two groups. The correlation analysis was conducted using Pearson’s test to analyze the correlation between the two instruments. When 0 < |r| ≤ 0.3, it indicates that the two variables are not correlated; when 0.3 < |r| ≤ 0.5, it indicates a low correlation; when 0.5 < |r| ≤ 0.8, it indicates a moderate correlation; and when 0.8 < |r| ≤ 1.0, it indicates a strong correlation. A difference was considered statistically significant when *p* < 0.05.

## 3. Results

### 3.1. Patient Demographics

The differences between normal individuals and patients with keratoconus were evaluated by an independent sample *t*-test. The age of normal individuals was 25.5 ± 5.5 years, with a male proportion of 52.4%. The age of patients with keratoconus was 23.5 ± 5.2 years, with a male proportion of 48%.

### 3.2. Analysis of BOSS Results

**Figure 2:**
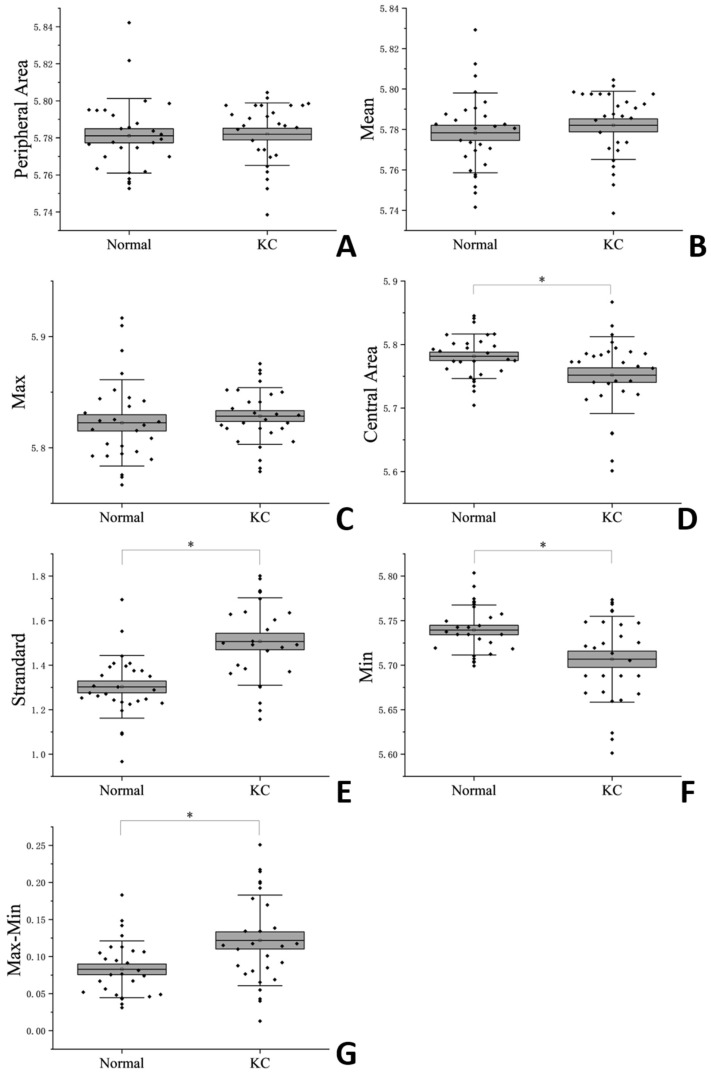
Corneal Brillouin Frequencies (**A**): Peripheral Area; (**B**): Mean; (**C**): Max; (**D**): Central Area; (**E**): Standard; (**F**): Max; (**G**): Max–Min. * indicate statistically significant differences between the normal eyes and the KC eyes (*p* < 0.05).

Table 1 and Figure 2 shows the results of the BOSS measurements among different regions. In the comparison of differences between normal eyes and keratoconus eyes, only the nasal inferior region showed a statistically significant difference (*p* = 0.004) among the eight regions. In the other seven regions, no statistically significant differences were observed (*p* > 0.05). The results after regional integration showed that the Brillouin frequency in the central region of keratoconus eyes was significantly lower than that in normal eyes (*p* = 0.029), while no statistically significant difference was observed in the peripheral region (*p* = 0.781). There was no difference in the Mean (*p* = 0.452) and Max (*p* = 0.487) values, but the Min (*p* = 0.003), Standard (*p* = 0.000) and Max–Min (*p* = 0.006) values showed differences.

### 3.3. Analysis of Corvis ST Results

**Figure 3:**
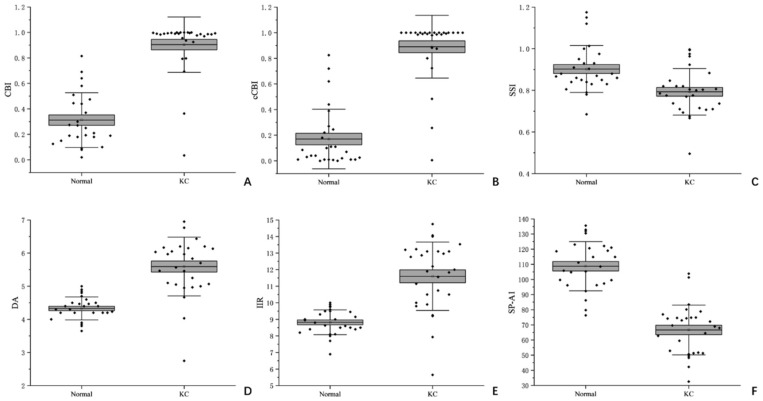
Box plots of Corvis ST parameters for normal eyes and keratoconus eyes (**A**): CBI; (**B**): cCBI; (**C**): SSI; (**D**): DA; (**E**): IIR; (**F**): SP-A1.

The descriptive statistics of Corvis ST data are shown in Table 2 and Figure 3. The corneal elasticity reflected by corneal biomechanical parameters in patients with keratoconus was generally softer than that in normal eyes. In comparison with normal eyes, significant statistical differences were observed in six parameters, namely CBI, CCBI, SSI, DA, IIR and SPA1 (*p* < 0.001).

### 3.4. Correlation Analysis Between BOSS and Corvis ST

**Figure 4:**
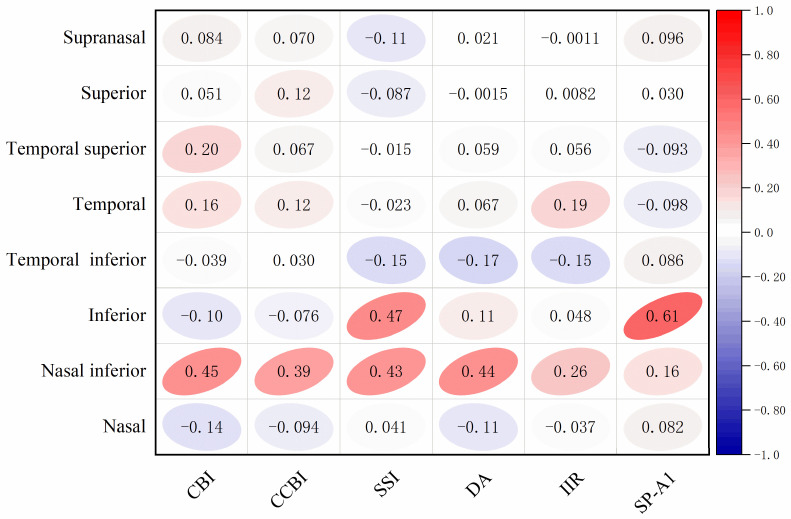
Correlation analysis of Brillouin frequencies in each region and Corvis ST parameters.

After normalizing the parameters of the Brillouin microscopy and Corvis ST, a correlation analysis was conducted (Figure 4). It was found that the Brillouin frequency inferior area had a moderate correlation with SSI (r = 0.47, *p* = 0.012) and SP-A1 (r = 0.61, *p* = 0.001). There was a certain correlation between the nasal inferior area and CBI (r = 0.45, *p* = 0.015), CCBI (r = 0.39, *p* = 0.038), SSI (r = 0.43, *p* = 0.023), and DA (r = 0.44, *p* = 0.019).

## 4. Discussion

The biomechanical properties of the cornea are of great significance in the diagnosis of keratoconus. Previous studies have shown that the local biomechanical properties of the cone zone in keratoconus are weakened, while the biomechanical properties outside the cone zone are normal [23]. This uneven weakening of biomechanical properties and the focal reduction in the elastic modulus can trigger biomechanical decompensation cycles driven by biomechanical asymmetry, thereby leading to the occurrence and development of the disease [24]. However, the biomechanical measurement methods currently used in clinical practice cannot assess regional differences, thereby hindering the development of personalized and precise treatment [9]. The results of the Brillouin microscopy in this study show that there are statistically significant differences in the Brillouin frequency shift between normal individuals and keratoconus patients in Central, Min, Standard, and Max–Min, indicating that the corneas of the keratoconus group are significantly softer. This has been confirmed in the overall Brillouin frequency changes observed in keratoconus [25]. There are significant differences in the minimum value, standard deviation, and maximum–minimum value between the two groups, which is consistent with the findings of Zhang et al. [26]. The overall and regional changes further confirm the feasibility of Brillouin microscopy in the diagnosis of keratoconus.

We increased the scanning resolution by using 10 points. Statistical analysis revealed that there were no differences in the entire cornea between normal individuals and patients with keratoconus. However, statistical differences were observed only at the points below the nasal area. The keratoconus lesion usually begins in the lower part of the cornea or the temporal inferior side [27,28]. The biomechanical properties of the cornea in this area are weakened, which, in contrast, makes the nasal side slightly stronger.

This result suggests that there may be regional differences in corneal biomechanical properties, especially in keratoconus, where the biomechanical changes in the lesion area may be more significant [8]. This regional difference may be related to the anatomical structure of the cornea [29], the distribution of collagen fibers [30,31], and the non-uniformity of lesion progression [24].

The research conducted by Riccardo Vinciguerra et al. also utilized Brillouin microscopy and Corvis ST to compare the corneal biomechanical properties of normal individuals and patients with keratoconus. Their findings revealed that Brillouin microscopy exhibited significant differences at multiple measurement points (including the superior, middle, and inferior regions), while Corvis ST demonstrated significant differences in multiple biomechanical parameters [32]. This is consistent with the results of our study, indicating that both devices can effectively distinguish normal corneas from keratoconus corneas, but there are differences in measurement sensitivity and specificity.

Corvis ST and Brillouin microscopy each have their own advantages and limitations in the diagnosis of keratoconus [33]. Corvis ST measures corneal biomechanical parameters through dynamic Scheimpflug imaging technology and has been proven effective in the diagnosis of expansive corneal diseases such as keratoconus in previous studies [34,35]. In this study, six parameters including CBI, CCBI, SSI, DA, IIR, and SPA1 were included. These parameters showed significant statistical differences between normal eyes and keratoconus eyes (*p* < 0.001), indicating their high sensitivity and specificity in the diagnosis of keratoconus. However, the measurement results of Corvis ST may be affected by the corneal shape and refractive status [36,37]. Moreover, the biomechanical parameters measured by Corvis ST rely on the measurement of biomechanical properties along a single axis and mainly reflect the overall characteristics of the cornea, but do not provide regional corneal biomechanical information [15]. Although the regional distribution of corneal biomechanical performance has been confirmed through various forms of in vitro experiments in animals [38,39,40], and regional changes in the response of corneal surfaces to pressure variations have been found in studies of isolated human eyes [41,42], current research on the regional differences in the biomechanical performance of in vivo corneas is still insufficient. Meanwhile, the improvement in the biomechanical performance of the corner after corneal cross-linking is not consistent [39,43], and the measurement results of Corvis ST cannot effectively guide the design of precise corneal cross-linking surgical plans. Brillouin microscopy measures the Brillouin frequency shift in the cornea to assess its biomechanical properties, offering higher spatial resolution and sensitivity to local lesions. It can detect subtle changes in corneal biomechanics [44] and can create a three-dimensional map of the entire cornea, providing more comprehensive information on corneal biomechanics. Therefore, it can evaluate local areas of the cornea, which is of great significance for diagnosing localized keratoconus [45,46]. Its aim is to measure the intrinsic material properties of the cornea, which are largely independent of the corneal thickness and intraocular pressure [47], and by understanding the local biomechanics of cells and tissues, it becomes a key to predicting cell fate and the pathogenesis of tissue diseases [48]. Meanwhile, Brillouin microscopy allows for the quantitative description of corneal mechanical changes after CXL surgery [49], enabling more precise assessment of the effect of CXL in terms of penetration depth and spatial accuracy, avoiding unnecessary side effects [50,51], and further guiding the personalized treatment of CXL.

Some studies have indicated that the combined use of multiple devices (such as Corvis ST and Pentacam) can enhance the diagnostic accuracy of keratoconus [35,38,52].

These studies have emphasized the significance of multimodal imaging techniques in the diagnosis of keratoconus. Although the longitudinal modulus measured by Brillouin microscopy is different from the traditional Young’s modulus [49], it cannot be directly compared with the results obtained from other detection devices [17]. Through the normalization of the results of the two detection methods, we found that the measurement results of Brillouin microscopy in the nasal area of the cornea have a certain correlation with the corneal biomechanical index (CBI), composite corneal biomechanical index (CCBI), stress–strain index (SSI), and deformation amplitude (DA) of Corvis ST. In addition, the measurement results in the nasal area of the cornea also show a certain correlation with the SSI and first flattening stiffness parameter (SP-A1) of Corvis ST (0.3 < r < 0.7). Previous studies by Lopes et al. have observed for a long time in KC patients that significant differences are found in the cone region of keratoconus compared with healthy corneas in the Brillouin frequency and SSI [45]. These results not only reveal the correlation with Corvis ST parameters, provide a new method for corneal biomechanical assessment, and offer potential application value for the diagnosis and treatment of corneal diseases, but also further prove that the combined use of multiple devices may provide more comprehensive information for the diagnosis of keratoconus [2].

However, our research also has certain limitations. This study did not include patients with intermittent keratoconus and failed to fully assess the differences in Brillouin frequency shifts among different stages of keratoconus [19]. It is still necessary to further explore whether the Brillouin microscope can detect subclinical differences in keratoconus and whether the individual inter-individual variability of Brillouin frequency shifts is related to the physiological conditions of the cornea. The influence of different detection times on the hydration state of the cornea also needs to be further investigated [53]. The acoustic properties of the cornea are extremely sensitive to its water content, and thus the deviation caused by different hydration levels cannot be completely excluded [53,54,55]. The spatial resolution advantage of the Brillouin microscope enables it to capture more precisely the biomechanical changes in different regions of the cornea. It reflects the elastic modulus of the cornea by measuring the Brillouin frequency shift and can effectively distinguish the biomechanical characteristics of normal corneas and keratoconus [46]. However, the long data acquisition time and the potentially harmful light dose during measurement, as well as the stability requirements of the equipment and operational techniques during the measurement process, all limit the wide application of this technology in clinical practice [56].

Brillouin microscopy enables high-resolution imaging of the elastic modulus of local regions of the cornea without contact or invasiveness, providing regional mechanical information that is difficult to obtain through traditional biomechanical detection techniques. This technology demonstrates significant added value in the precise diagnosis and treatment of corneal diseases. In terms of diagnosis, Brillouin microscopy can detect subtle changes in the local stiffness of the cornea (such as the temporal weakening of mechanics in the subclinical stage of keratoconus) early on, compensating for the lack of morphological examinations (such as Pentacam), and improving the sensitivity of disease screening. In follow-up and staging, its dynamic monitoring capability can quantify the regional differences in disease progression (such as the rate of mechanical attenuation around the cone body in keratoconus), assisting in the establishment of individualized intervention thresholds. For treatment response assessment, this technology can visually display the spatial distribution of the collagen cross-linking strength after corneal cross-linking (such as a more significant increase in modulus in the central area compared to the periphery), providing a basis for optimizing the light dose and riboflavin concentration gradients, while avoiding excessive treatment. Compared to traditional overall biomechanical detection (such as Corvis ST), the depth resolution and micrometer-level spatial resolution of Brillouin microscopy enable clinicians to identify “mechanical microfoci” lesions.

## 5. Conclusions

This study compared the application of Corvis ST and Brillouin microscopy in the diagnosis of keratoconus, revealing the advantages and limitations of the two devices in the measurement of corneal biomechanics. Corvis ST demonstrated higher sensitivity and specificity in the measurement of overall biomechanical parameters, while Brillouin microscopy had a higher resolution and sensitivity in the detection of local lesions. Moreover, this study also emphasized the regional differences in corneal biomechanics, suggesting that in clinical diagnosis, the biomechanical characteristics of different regions of the cornea should be comprehensively considered. At the same time, it was confirmed that in keratoconus patients, the regional biomechanical properties of the cornea change, especially in the nasal area and the lower part, and there is a certain correlation with the overall change in the corneal biomechanical properties.

## Figures and Tables

**Table 1:** The measurement results for the Brillouin frequency in different regions.

Regions	Calculation	Brillouin Frequency (GHz)		
		Normal Eyes	Keratoconus Eyes	*p*
Nasal up	(w1 + w2 + w3)/3	5.81 ± 0.03	5.80 ± 0.03	0.118
Superior	w1	5.78 ± 0.04	5.77 ± 0.05	0.266
Temporal up	(w1 + w7 + w8)/3	5.78 ± 0.02	5.77 ± 0.04	0.618
Temporary	w7	5.78 ± 0.04	5.78 ± 0.05	0.701
Temporal down	(w5 + w6 + w7)/3	5.78 ± 0.03	5.80 ± 0.03	0.084
Inferior	w5	5.77 ± 0.02	5.78 ± 0.04	0.184
Nasal down	(w3 + w4 + w5)/3	5.77 ± 0.03	5.80 ± 0.04	0.004
Nasal side	w3	5.78 ± 0.04	5.78 ± 0.03	0.632
Peripheral	(w1 + w2 + w3 + w4 + w5 + w6 + w7 + w8)/8	5.78 ± 0.02	5.78 ± 0.02	0.781
Central	System value	5.78 ± 0.04	5.75 ± 0.06	0.029
Mean	System value	5.78 ± 0.02	5.78 ± 0.02	0.452
Min	System value	5.74 ± 0.03	5.71 ± 0.05	0.003
Max	System value	5.82 ± 0.04	5.83 ± 0.03	0.487
Standard	System value	1.30 ± 0.14	1.51 ± 0.20	0.000
Max-Min	System value	0.08 ± 0.04	0.12 ± 0.06	0.006

**Table 2:** Descriptive statistics results of Corvis ST data.

Parameter	Normal Eyes	Keratoconus Eyes	*p*
CBI	0.31 ± 0.22	0.90 ± 0.22	<0.001
CCBI	0.17 ± 0.23	0.89 ± 0.25	<0.001
SSI	0.90 ± 0.11	0.78 ± 0.11	<0.001
DA (mm)	4.33 ± 0.35	5.61 ± 0.90	<0.001
IIR (mm^−1^)	8.82 ± 0.75	11.64 ± 2.09	<0.001
SP-A1 (mmHg/mm)	108.74 ± 16.23	66.34 ± 16.69	<0.001

## Data Availability

The original contributions presented in the study are included in the article; further inquiries can be directed to the corresponding authors.
